# Spontaneous regression of advanced hepatocellular carcinoma: a case report

**DOI:** 10.4076/1757-1626-2-6251

**Published:** 2009-08-07

**Authors:** Chuan-Yuan Hsu, Po-Lin Sun, Hong-Cheng Chang, Daw-Shyong Perng, Yaw-Sen Chen

**Affiliations:** 1 Department of Gastroenterology & Hepatology, E-Da Hospital, I-Shou UniversityNo 1, Yi-Da Road, Jiau-shu Tsuen, Yan-chau Shiang, Kaohsiung CountyTaiwan, R.O.C.; 2 Department of Diagnostic Radiology, E-Da Hospital, I-Shou UniversityNo 1, Yi-Da Road, Jiau-shu Tsuen, Yan-chau Shiang, Kaohsiung CountyTaiwan, R.O.C.; 3 Department of Pathology, E-Da Hospital, I-Shou UniversityNo 1, Yi-Da Road, Jiau-shu Tsuen, Yan-chau Shiang, Kaohsiung CountyTaiwan, R.O.C.; 4 Department of General Surgery, E-Da Hospital, I-Shou UniversityNo 1, Yi-Da Road, Jiau-shu Tsuen, Yan-chau Shiang, Kaohsiung CountyTaiwan, R.O.C.

## Abstract

Spontaneous regression of advanced hepatocellular carcinoma is extremely rare. A 66-year-old Taiwanese male patient with liver cirrhosis related to chronic hepatitis C presented with hepatocellular carcinoma with portal vein thrombosis. At first, he refused curative therapy, except for silymarin medicine. Spontaneous regression of hepatocellular carcinoma occurred with a decline in tumour size and tumour marker in imaging studies. The patient agreed to undergo surgery approximately 14 months after presentation because of no further decrease in tumour size and an increase in tumour marker in the imaging studies. The resected tumour was hepatocellular carcinoma with portal vein thromboses. Presently, the patient is alive and in good condition without any symptoms or tumour recurrence. We concluded that this was a rare case of spontaneous regression of advanced hepatocellular carcinoma.

## Introduction

Primary hepatocellular carcinoma (HCC) is one of the most common cancers, especially in Asia and Africa. It has a high mortality rate and increasing incidence in the world. Advanced HCC has a poor prognosis, and untreated tumors show a rapidly fatal outcome, with a median survival of 1.6 months [1]. However, spontaneous regression of HCC is very rare and its mechanism is unclear [2]. There is no curative therapy for advanced HCC [3]. Therefore, it has been suggested that HCC cases be given more attention. Here we present a rare case of a patient with spontaneous regression of advanced HCC without any curative therapy.

## Case presentation

**Figure 1.**
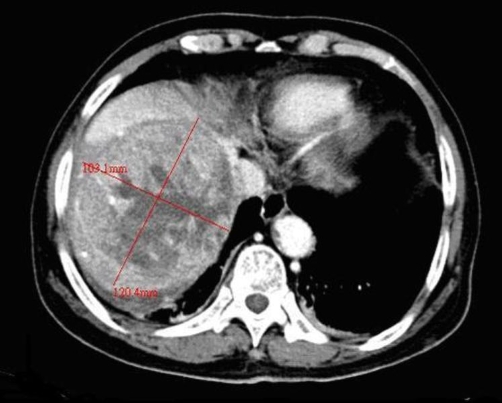
CT scan of a large well-defined mosaic mass lesion in right hepatic lobe with early contrast medium filling and washout with central necrosis, measuring about 12 cm in diameter.

In March 2006, a 66-year-old Taiwanese male patient complained of having upper right quadrant discomfort for about 10 days. The patient also suffered from poor appetite and fatigue. His past history was significant with a traffic accident and right leg amputation about 30 years ago. The patient had no known exposure to chemicals, did not drink, and smoked one pack per day for about 30 years. He had gout disease and took analgesic medicine. Ultrasonography in our outpatient department revealed a large tumour with central necrosis, measuring about 11 cm in diameter, occupying the right lobe and left hydronephrosis. Gastroscopy revealed pathology of active gastric ulcers without *Helicobacter pylori* infection. In laboratory studies, the patient had a high serum α-fetoprotein (AFP) level of 4280 ng/ml (normal range, 0-13.4 ng/ml) and a positive hepatitis C antibody. We subsequently performed computed tomography (CT) of the liver, which revealed a large tumour with central necrosis, about 12 cm in diameter, located in segments 7 and 8 of the right lobe (Figure 1), along with left hydronephrosis and a ureter stone.

**Figure 2.**
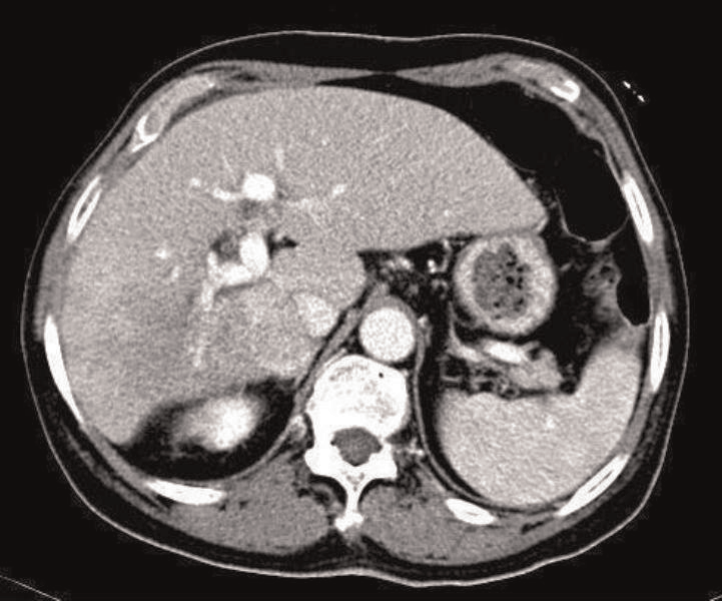
Disturbance in contrast on posterior branches of the right portal vein with portal vein tumour thrombosis.

The CT scan also revealed tumour thrombus in the posterior branches of the right portal vein (Figure 2).

According to Barcelona-Clinic Liver Cancer classification, we made the diagnosis of HCC with portal vein thrombosis [3]. However, the patient refused any invasive therapy and examination for HCC, such as a biopsy, surgery, radiofrequency ablation, or transcatheter arterial chemoembolization (TACE). The patient agreed only to treatment for left hydronephrosis and the ureter stone, and the regular, oral administration of silymarin medicine of 450 mg per day.

The patient was regularly followed-up in our outpatient department with ultrasonography and examination of serum α-fetoprotein levels. The tumour size gradually decreased, and the α-fetoprotein level slowly declined (Table 1) until December 2006. The patient did not take any herbs or other curative or palliative therapy during the HCC shrinkage.

**Table 1.** Serum α-fetoprotein (normal range 0-13.4 ng/ml)

	Mar-2006	Nov-2006	Dec-2006	Feb-2007	May-2007	Jun-2007	Nov-2008
AFP	4280	717	546	724	1209	34.8	11.5

**Figure 3.**
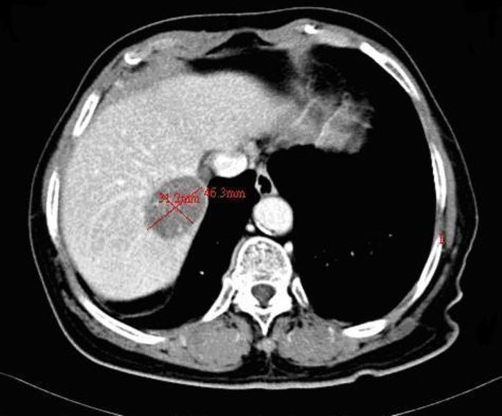
Right hepatic tumour shrinkage to about 4.6 cm in diameter.

CT scan of the liver was performed again and revealed HCC regression, with tumour size reduced to about 4.6 cm in diameter, and portal vein thrombosis (Figure 3). Silymarin 450 mg daily was the only regularly prescribed medicine during HCC reduction.

**Figure 4.**
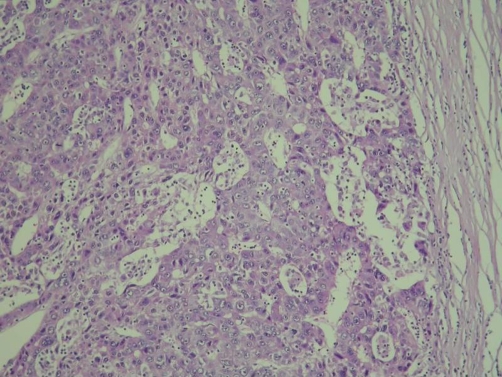
Histology of viable tumour cells, surrounded by thick fibrous capsule, in a moderately differentiated hepatocellular carcinoma (HCC) with trabecular and focal pseudoglandular pattern.

**Figure 5.**
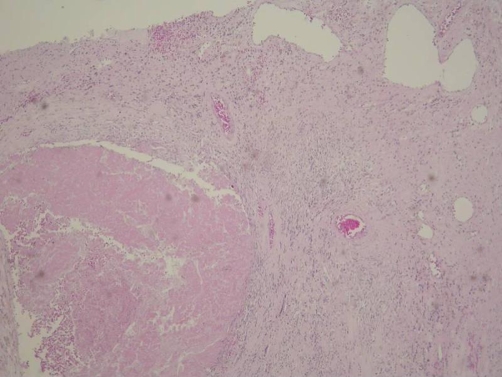
Necrotic tumour thrombi in right posterior branch of portal vein.

Nevertheless, the serum α-fetoprotein level increased gradually from December 2006 (Table 1), and the tumour size did not contract persistently as shown by abdominal echo. We talked to the patient about his condition and recommended surgery. The patient agreed to the surgery, and we scheduled surgery for May 2007. CT scan of the liver was performed prior to the surgery, revealing an HCC size similar to the previous readings and portal vein thrombosis. A total right lobectomy and cholecystectomy were performed. The resected specimen in fresh state was a piece of liver tissue, measuring 14.5 × 7.5 × 6.2 cm in size; 330 g in weight; and brown with an uneven surface macroscopically. On cuts, an ill-defined tumour, measuring 6 × 4 × 3.5 cm in size, was observed at the subcapsular area. The tumour was encapsulated, yellow, and soft with a marked necrosis. Multiple satellite nodules, measuring up to 1.7 × 1.5 × 1.5 cm in size, were observed. The surrounding liver tissue was brown and cirrhotic. Histologically, the viable tumour cells appeared as moderately differentiated HCC with a trabecular and focal pseudoglandular pattern (Figure 4). Tumour thrombi were located in the right posterior branch of the portal vein (Figure 5). The tumour had central coagulative necrosis, similar to a post-transcatheter arterial chemoembolization (TACE) effect. The tumour extended into the subcapsular area and was encapsulated by a thick, fibrous capsule. The main tumour was surrounded by satellite nodules, which were histologically similar to the main tumour. The background liver had a non-neoplastic histology of chronic hepatitis C with cirrhosis.

The postoperative course of this patient was smooth. The serum α-fetoprotein level declined rapidly after the operation, and it is currently at the normal level (Table 1). Twenty months after the surgery, the patient is doing well and has no signs of tumour recurrence.

## Discussion

Spontaneous regression has been defined by Everson and Cole as the partial or complete involution of a malignant tumour without application of a specific therapy [2]. Spontaneous regression has been reported in a group of malignant diseases, including HCC. However renal cancer, neuroblastoma, and malignant melanoma are the main types of tumours that have shown spontaneous regression [4]. Spontaneous regression of HCC is a rare phenomenon. Few reports of spontaneous regression of HCC have been published in the English medical literature up to 2008 [5].

The pathogenesis of spontaneous regression is unclear. Possible mechanisms proposed in the literature include abstinence from alcohol consumption, taking herbal medicine, arterioportal shunting, high fever, immune system, androgen therapy, subintimal vascular injury by angiographic procedures, rapid growth of tumour, gastrointestinal bleeding, administration of vitamin K, life style modification, poor vascular circulation in cirrhosis, vascular thromboses, and thick capsule [5]. Even on closer examination of published cases on spontaneous regression of HCC, precise conclusions about the mechanisms are difficult to derive. However, several authors have suggested the possibility of an alternation in the vascular supply and changes in the immune system.

The histological findings of our patient, including the tumour necrosis and the thick fibrous capsule, are similar to findings of a post-TACE effect [6]. The inflammatory cells, which infiltrated into the fibrous capsule and inside of tumour, also suggest that the inflammatory reaction in the patient was long-standing. Namely, it can be concluded that changes in the immune system of the patient were induced by vascular disturbance. The changes in the vascular supply as well as in the immune system demonstrate good anticancer effects.

Grossmann et al. reported a spontaneous regression of HCC after the abstinence and administration of silymarin [7]. Silymarin is a bioflavonoid antioxidant, and it is the main constituent of *Silybum marianum*. Silymarin is composed of silybin (also called silybinin, silybin, or silibinin) with smaller amounts of other stereoisomers such as isosilybin, dihydrosilybin, sildianin, and silychristin [8]. Silymarin has a strong antioxidant property and is capable of scavenging both free radicals and reactive oxygen species [9]. In addition to silymarin’s antioxidant effect, its anticancer effect *in vitro* has been certified by Lah et al [1]. Although silymarin has been used for centuries, its anticancer mechanism in HCC is still unknown. In previous studies, possible mechanisms may have included inhibition of extracellular signal-regulated kinase 1/2 phosporylation [11]; down-regulation expression of cyclooxygenase-2 and attenuation hyperlipidemia [12]; inhibition of cytochrome P450 1A1 catalytic activity [13]; and inhibition of proliferation and induction of apoptosis [14]. Our patient took silymarin regularly prior to surgery, and he did not take any other herbs or medicines. Some cases of regression have been associated with some herbal medicines, but there is no conclusive evidence. However, thus far, we have not clearly understood the effect and mechanism of silymarin in HCC. Further studies are needed to confirm these findings and to more fully characterise the factors affecting HCC regression.

## Conclusions

In summary, we report spontaneous regression of advanced HCC in the case of a 66-year-old male patient. Even after a literature review, spontaneous regression of HCC are still unclear. It is possible that circulatory disturbance and immune mechanisms may be major pathways. Silymarin should be further investigated for its anticancer effect. In conclusion, it is likely that treatment of HCC in the future will include a multimodal therapeutic method.

## Abbreviations

AFP: α-fetoprotein
CT: Computed tomography
HCC: Hepatocellular carcinoma
TACE: Transcatheter arterial chemoembolization
